# A cold seep triggered by a hot ridge subduction

**DOI:** 10.1038/s41598-021-00414-3

**Published:** 2021-10-22

**Authors:** Lucía Villar-Muñoz, Masataka Kinoshita, Joaquim P. Bento, Ivan Vargas-Cordero, Eduardo Contreras-Reyes, Umberta Tinivella, Michela Giustiniani, Natsue Abe, Ryo Anma, Yuji Orihashi, Hikaru Iwamori, Tomoaki Nishikawa, Eugenio Andres Veloso, Satoru Haraguchi

**Affiliations:** 1 Departamento de Geofísica, Facultad de Ciencias Físicas y Matemáticas, Universidad de Chile, Santiago, Chile; 2 Earthquake Research Institute, The University of Tokyo, Tokyo, 113-0032 Japan; 3 Escuela de Ciencias del Mar, Pontificia Universidad Católica de Valparaíso, Valparaíso, Chile; 4 Istituto Nazionale di Oceanografia e di Geofisica Sperimentale - OGS, 34010 Trieste, Italy; 5 Mantle Drilling Promotion Office, MarE3, Japan Agency for Marine-Earth Science and Technology (JAMSTEC), Yokohama, Japan; 6 Graduate School of Technology, Industrial and Social Sciences, Tokushima University, Tokushima, Japan; 7 Global Environment and Disaster Prevention, Graduate School of Science and Engineering, Hirosaki University, Hirosaki, Japan; 8 Disaster Prevention Research Institute, Kyoto University, Kyoto, Japan; 9 Andean Geothermal Center of Excellence (CEGA), Santiago, Chile

**Keywords:** Geology, Geophysics, Volcanology, Natural gas, Geothermal energy, Natural hazards, Solid Earth sciences

## Abstract

The Chile Triple Junction, where the hot active spreading centre of the Chile Rise system subducts beneath the South American plate, offers a unique opportunity to understand the influence of the anomalous thermal regime on an otherwise cold continental margin. Integrated analysis of various geophysical and geological datasets, such as bathymetry, heat flow measured directly by thermal probes and calculated from gas hydrate distribution limits, thermal conductivities, and piston cores, have improved the knowledge about the hydrogeological system. In addition, rock dredging has evidenced the volcanism associated with ridge subduction. Here, we argue that the localized high heat flow over the toe of the accretionary prism results from fluid advection promoted by pressure-driven discharge (i.e., dewatering/discharge caused by horizontal compression of accreted sediments) as reported previously. However, by computing the new heat flow values with legacy data in the study area, we raise the assumption that these anomalous heat flow values are also promoted by the eastern flank of the currently subducting Chile Rise. Part of the rift axis is located just below the toe of the wedge, where active deformation and vigorous fluid advection are most intense, enhanced by the proximity of the young volcanic chain. Our results provide valuable information to current and future studies related to hydrothermal circulation, seismicity, volcanism, gas hydrate stability, and fluid venting in this natural laboratory.

## Introduction

The Chile Triple Junction (CTJ) of the oceanic Nazca, Antarctic, and continental South American plates is the “hottest subduction zone” on Earth, being the only modern site where an active oceanic spreading ridge, the Chile Rise (CR), is currently subducting beneath the South American plate^1,2^, in a perpendicular Ridge-Trench-Trench configuration. Due to the high heat flow and dehydration in the shallow part of the subduction zone, the CTJ offers a unique opportunity to observe and analyse different geological factors influenced by its anomalous thermal regime.

The subduction of the CR leaves distinctive features in the geological records of the overriding South American plate, such as: (a) regional metamorphism and high thermal gradient^3–7^; (b) a hiatus in arc magmatism^8,9^; (c) near trench magmatism^10–18^; (d) subduction erosion process^1,14,19^; (e) hydrothermal circulation^7,20,21^; (f) continuous tectonic uplift of the Andes^14,22^; (g) wedge shortening^23^; (h) slab window^2,24–26^; (i) ophiolite obduction^10,27–31^; and (l) a distinct continuous, shallow and strong bottom-simulating reflector (BSR)^6^. The latter marks the base of the gas hydrate stability zone, and exhibits a significant decrease in gas hydrate concentration (10% of the total volume) in the vicinity of the CTJ^32^.

In order to better understand the factors affecting the thermal regime of the ridge subduction zone, we analyse the previous BSR-derived^6^ and directly measured heat flow data^4,5^ near the CTJ, the new heat flow data measured (heat flow piston core) right above the CTJ and a new estimate of the BSR-based heat flow from two seismic reflection profiles collected during the MR18-06 Leg2 cruise in 2019. We combine new high-quality multibeam bathymetric data acquired during this cruise with the legacy data. The bathymetric data is used to infer the seafloor morphology and identify distinctive features related to ridge volcanism and faulting, in conjunction with rock information derived from dredge deployments at the CTJ. We discuss indications for young volcanism at the toe of the accretionary prism in the vicinity of the CTJ.

Acquisition of heat flow data in situ would provide new insights into the deformation of the CR just prior to the collision with the forearc of the continental South American plate. These data, together with new information on the hydrogeological system and volcanism associated with ridge-subduction, may contain valuable information for current and future studies related to hydrothermal circulation, seismicity, volcanism, gas hydrate stability, and fluid venting. However, despite the importance of knowing the heat flow in the CTJ, only a few direct heat flow measurements have been performed in this area, mainly due to the high cost of implementation and research vessel availability. Thus, the direct heat flow measurements obtained in this study provide new insights into the regional thermal regime, and also allows us to compare for the first time the differences between direct measurements and BSR-derived estimations just above the CTJ.

### Geological and geothermal settings

**Figure 1:**
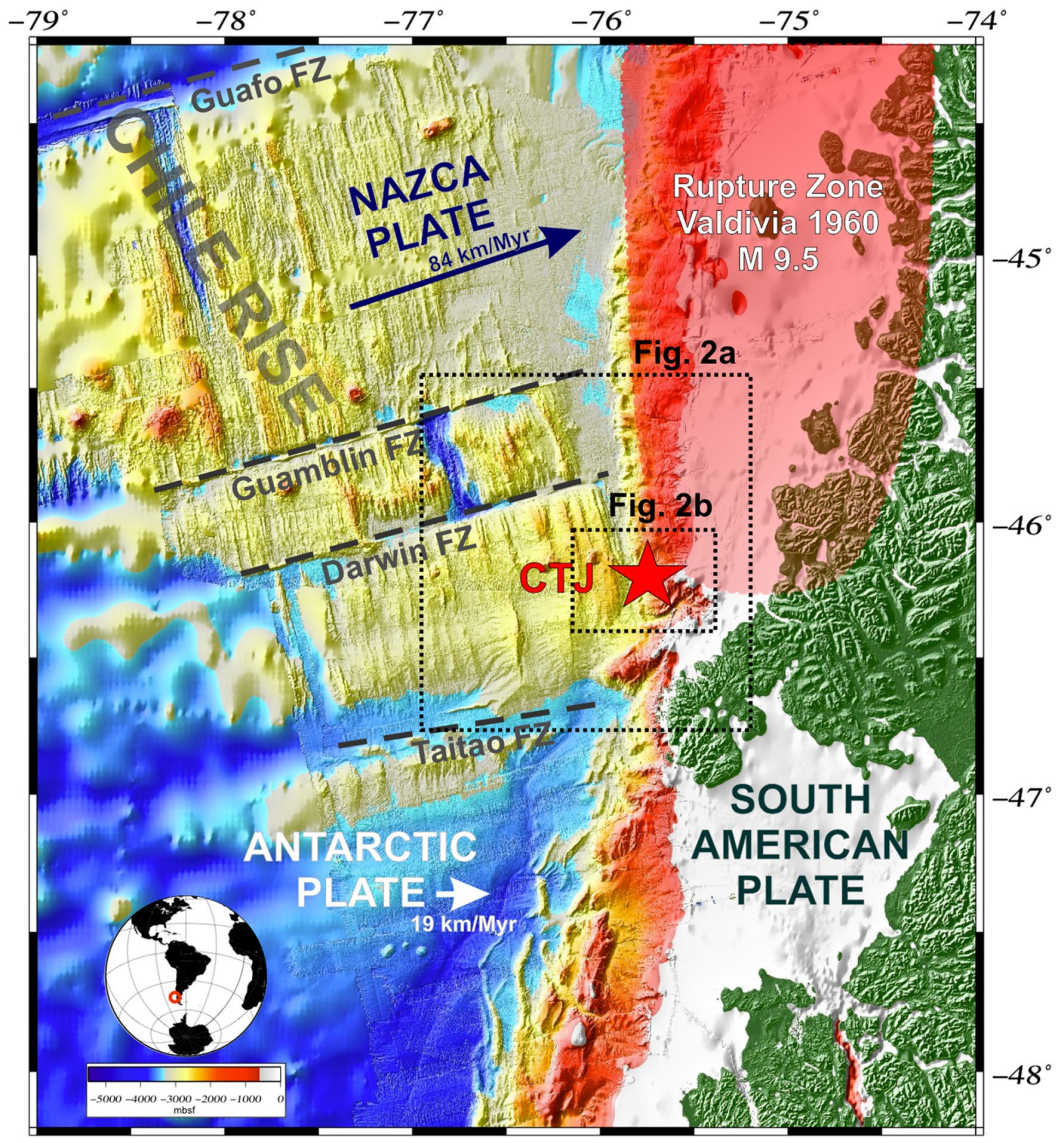
Location map of the study area offshore Taitao Peninsula. The bathymetry is based on GEBCO_2019 Grid (http://www.gebco.net). Tectonic setting of the Nazca, Antarctic, and South America plates: dashed grey lines show the main Fracture Zones (FZ), red star marks the triple junction of the plates (CTJ), and the red area delimits the rupture zone of the Valdivia’s mega-earthquake. Dashed rectangles correspond to Figs. 2a,b. Figure generated using GMT—v6.2.0 (https://www.generic-mapping-tools.org/).

The CTJ is located at ~ 46.2° S and corresponds to the intersection of three tectonic plates: Nazca (NP), Antarctic (AP), and South American (SA)^5,19,33^. Here, the CR, the active spreading centre that creates the oceanic AP and NP, subducts beneath the continental SA plate (Fig. 1). The subduction of the CR started ∼14 million years (Ma) near Madre de Dios Island and then migrated northwards to its present location north of the Taitao Peninsula^1,34^.

North of the CTJ, the NP subducts beneath the SA plate in an ENE direction at an average rate of ~ 84 mm/y north of the CTJ^35,36^, and the AP is subducting in an ESE direction at ~ 19 mm/y south of the CTJ^37^. The CR spreading rate has been estimated at ~ 70 mm/y over the past 5 Ma^38^.

This region is characterised by subduction erosion in the CTJ (i.e. the loss of the crust of the overriding plate) and subduction accretion processes south and north of the CTJ (i.e. the transfer of material from the subducting plate into the overriding plate)^1,23^. The tectonic evolution of the margin begins from subduction erosion (5–5.3 to 1.5–1.6 Ma) followed by subduction accretion (1.5–1.6 Ma^14^).^39^ argued that the current subduction accretion along the pre-subduction segment is strongly related to a dramatic post-glacial increase in trench sediment supply. The collision zone between the CR and SA is characterized by active subduction erosion, where most of the forearc has been removed by the subducting CR. In contrast, in the post-collision zone, a large accretionary complex has been rebuilt further south by sediment offscraping and accretion during the slow subduction of the AP^1,19,23,40^. At the CTJ, the toe of the slope is currently undergoing uplifting as the spreading centre is subducted, reducing the pressure within the sediments^20^.

Near the CTJ, the accretionary prism made of accumulated sediments contains carbon that is converted to methane in a regime of very high heat flow and intense rock deformation enabling vigorous fluid migration. Here, gas hydrate (identified through the BSR) has a concentration that reaches up to 10% of the total rock volume, and a large amount of hydrate and free gas was estimated at about 7.21 × 10^11^ m^3^ and 4.1 × 10^10^ m^3^, respectively^7^.

Moreover, the location of the BSR close to the CTJ is surrounding the area where the water depth is about 2000 m; BSR depth is placed within the shallow sediments ranging from 90 to 280 m below the seafloor (mbsf), and it is recognized as a strong and continuous reflector over the entire area^6^.

Tectonically, the area of Southern Chile along the convergent margin is an active region characterised by high deformation rates, uplift and subsidence, and numerous earthquakes. This region is periodically affected by devastating earthquakes of Mw > 8^41^, including the strongest earthquake ever recorded -the great 1960 Mw 9.5 Valdivia earthquake^42^. This event started to rupture further north at 38° S and the rupture propagated southwards to the CTJ (^43^; see red area in Fig. 1). However, south of the CTJ, seismicity levels are much lower, reflecting the assimilation of the slowly subducting hot-new crust AP^44^.

**Figure 2:**
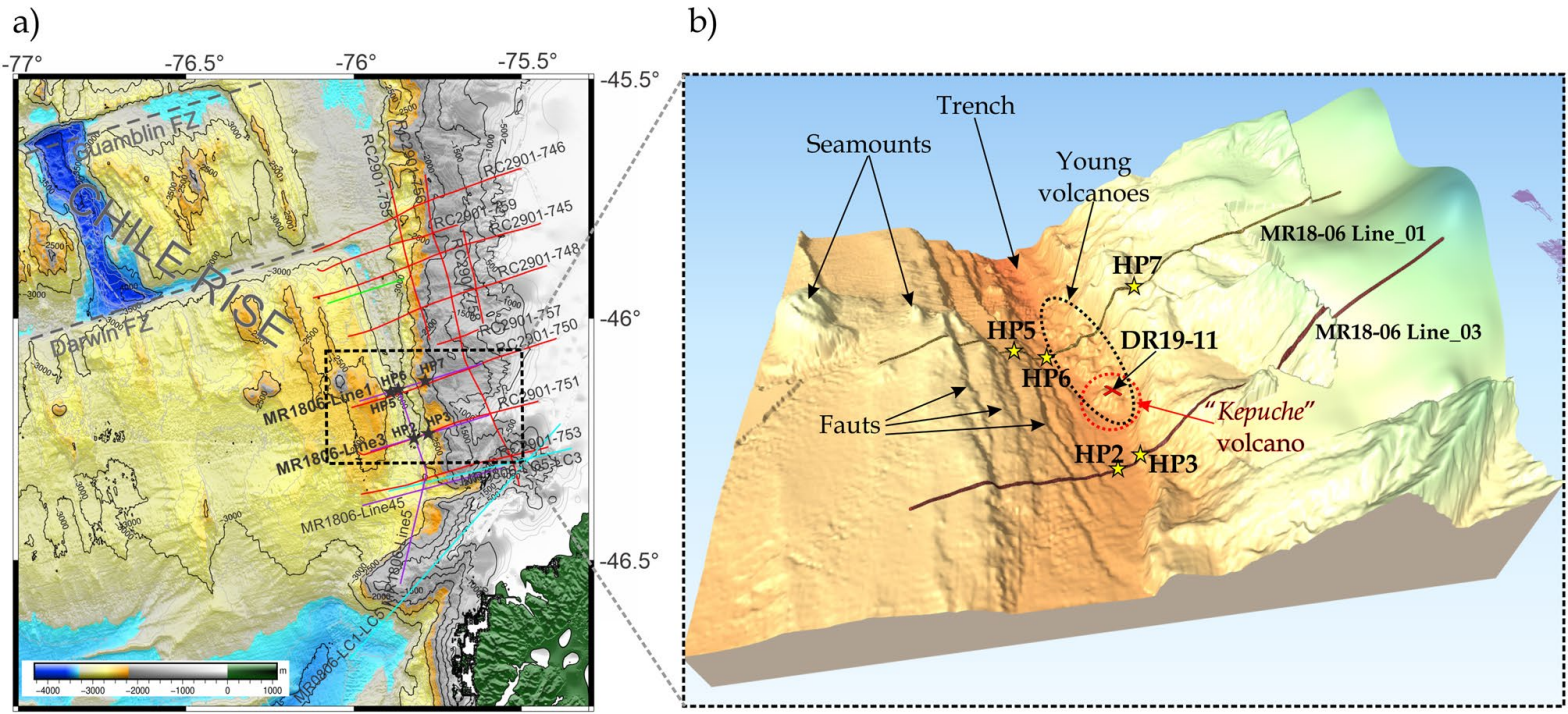
Database map: (**a**) Locations of the new single-channel seismic profiles (bold letters, purple lines) and heat probes measurements (black stars) showed in this study. The other profiles (red, green and sky blue lines) correspond to the database already published and adopted to create the bathymetry grids (based on GEBCO_2019 Grid) and the heat flow compilation reported in Fig. 8. Figure generated using GMT–v6.2.0 (https://www.generic-mapping-tools.org/). (**b**) 3-D projection of the high resolution bathymetry collected during MR18-06, showing the location of the two seismic profiles analysed in this study (solid lines), as well as show heat probes piston core measurements (yellow stars) and dredge sampling (red cross), dashed lines correspond to the young volcanic chain (black) and the “*Kepuche*'' volcano (red). Figure generated using QGIS-v3.16.8 (https://www.qgis.org/en/site/).

At the landward side of the trench (lower continental slope), heat flow values have been obtained with both methods, direct (heat probes) and indirect (BSR-derived), along the accretionary prism near the CTJ since the eighties (e.g.,^4–6,45^). It should be noted that most of the direct heat probe data were measured close to the Line RC2901-745 seismic profile (~ 45.8° S, Fig. 2) and corresponds to shallow heat flow data < 50 mbsf^4^. Moreover, the BSR-derived heat flow between the Darwin Fracture Zone (FZ) and the CTJ shows a heat flow high anomaly (~ 200 mW/m^2^ in average) above the subducting spreading ridge that may generate a powerful methane source at depth in the subduction zone. ^6^showed evidence that the heat flow is much greater south of the Darwin FZ than those indirect measurements to the north, reaching peak values of almost 300 mW/m^2^ at the seaward end of the seismic profile RC2901-745, where the eastern flank of the CR is today subducting. In the study area, maximum values occur at the toe of the accretionary prism, where active deformation^23^, and vigorous fluid flow, such as cold seeps, occur^46,47^.

In addition, the heat flow values along the profile RC2901-756 (see location in Fig. 2a) shows that heat flow along the strike of the forearc is on average ~ 200 mW/m^2^ on the lower forearc slope. However, the average heat flow in the overriding SA plate is not as high as thermal and subsidence models (~ 300 mW/m^2^ ^48^). The deviations of heat flow intensities have been attributed to the cooling effect of hydrothermal circulation through normal fault plate-bending related in the subducting NP^49^. Thus, this process reduces the thermal anomaly in the overriding plate as an exponential decay in heat flow with increasing subducting plate age (see Fig. 8 in^6^).

## Results

### Bathymetry

**Figure 3:**
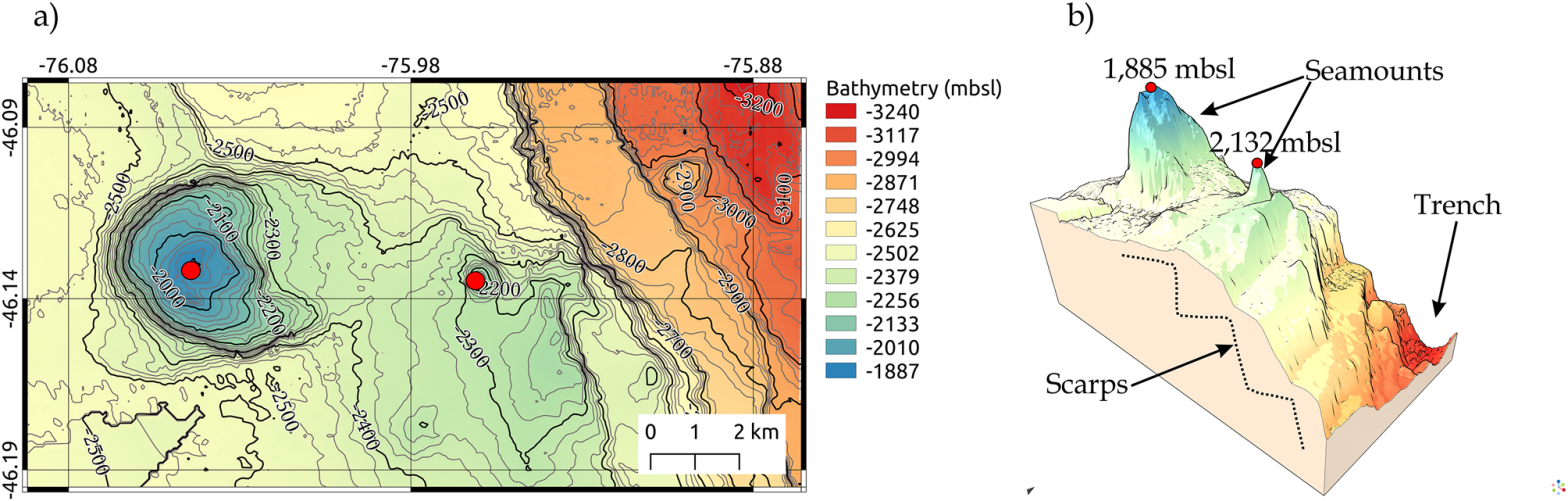
Regional setting of the seamounts near to the CTJ. Bathymetry collected during the expedition MR08-06 and MR18-06 Leg2. (**a**) Contour map of the study area and the most important seamounts identified in the oceanic crust. The red dots correspond to the summit in meters below the sea level (mbsl); (**b**) 3-D view of the identified seamounts and scarp-morphology of the west axial rift (dashed lines). Figures generated using QGIS-v3.16.8 (https://www.qgis.org/en/site/).

The newly high-resolution bathymetric data collected during the MR18-06 campaign allowed us to identify morphological features in and around the axial valley near the CTJ. The bathymetry shows that the oceanic crust to the west of the axial valley is rather flat, with a large (∼624 m high from its lower base) single feature that can be observed corresponding to a prominent seamount (western part in Fig. 3a) about 10 km west of the trench and a small feature in the direction of the trench, which also appears to be a small seamount of ∼147 m high. The large seamount appears to be in line with other small bathymetric hills, in an apparent WSW-ENE direction towards the centre of the axial valley, somewhat subparallel to the Darwin FZ. The central valley is bounded on the western flank by a series of east-facing scarps a few hundred meters high, apparently arranged in a stepped geometry with a series of morphologic relief ramps (Fig. 3b) connecting them northward. On the eastern part, the scarps are west-facing and less common compared to the western side of the axial valley. These scarps have previously been interpreted as normal faults (e.g.,^23^), and most likely related to axial valley propagation processes in the CTJ area (e.g.,^2^).

**Figure 4:**
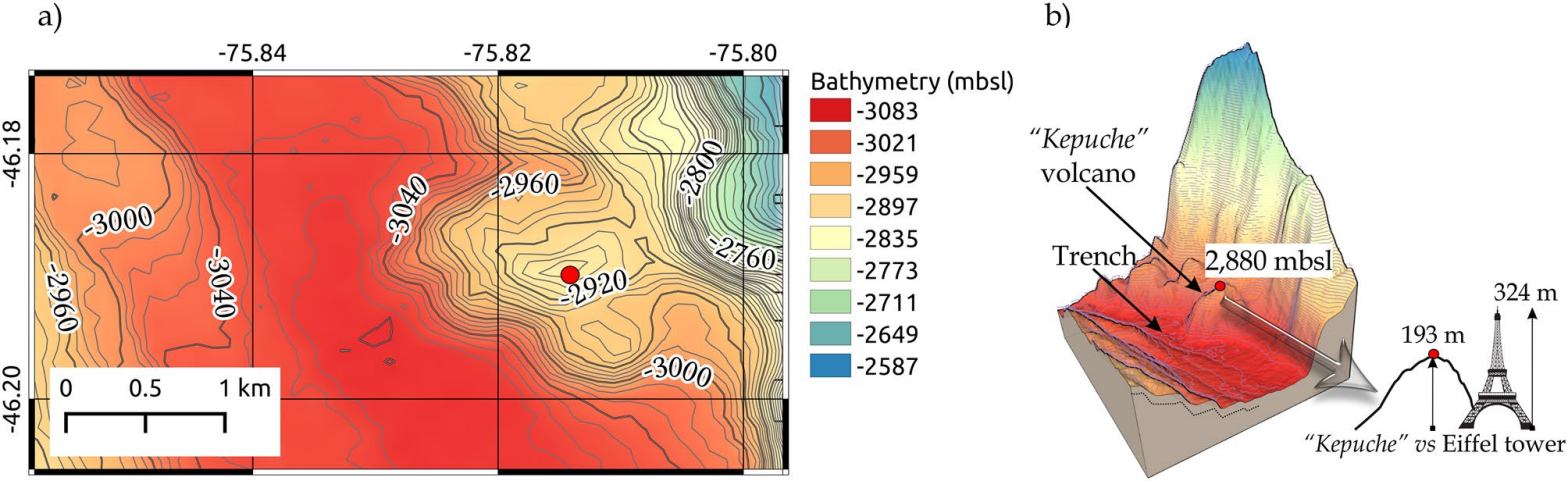
Regional setting of the area between the trench and the toe of the wedge. Bathymetry collected during the expedition MR08-06 and MR18-06 Leg2. (**a**) Contour map of the study area and the southern volcano. The red dot corresponds to the summit in meters below the sea level (mbsl); (**b**) 3-D zoom-in of the southern volcano, named “*Kepuche*”, the figures in the right corner represent an illustrative comparison of the height of the young volcano to the Eiffel tower. See text for more details. Figures generated using QGIS-v3.16.8 (https://www.qgis.org/en/site/).

Seaward of the toe of the accretionary wedge and trench, we observed several bathymetric elevations (indicated in Fig. 2b), considered to be young volcanoes as suggested by recovered dredge rocks (see further). The southernmost volcano (dashed red circle in Fig. 2b), where the dredge sampling was performed, has a height of approximately 193 m high from its lower base (Fig. 4a,b), here on referred to as the "*Kepuche*" volcano.

### Dredge

A fresh glassy basalt was sampled by dredging conducted at one of several axial volcanoes recognized in a detailed topographic survey by^50^. For details see Supplementary Information I.

### BSR-derived heat flow

**Figure 5:**
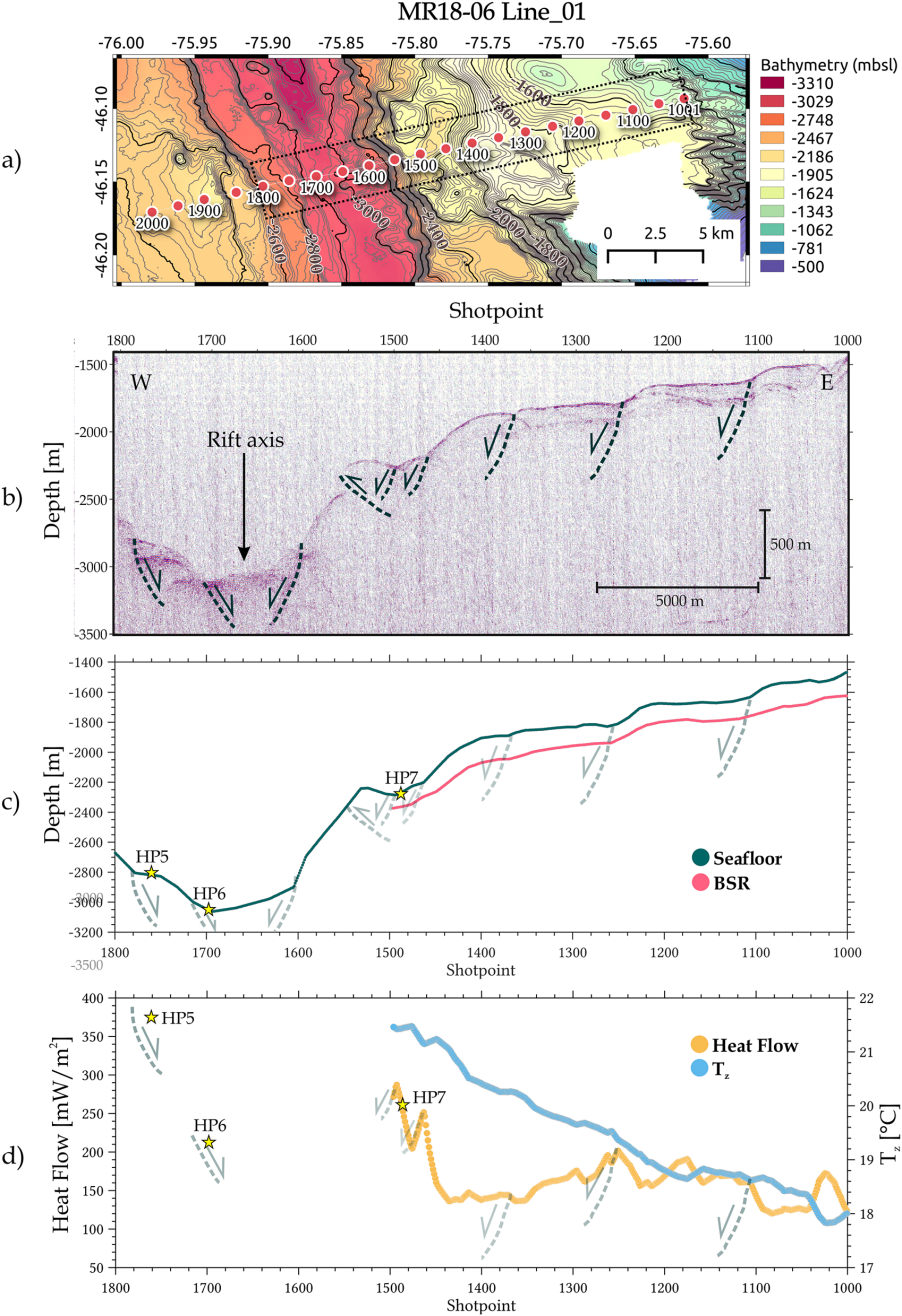
BSR-derived heat flow results from the MR18-06-Line_01 profile: (**a**) location of the seismic profile, red dots indicate the shotpoint values of the Line_01. Dashed rectangle indicates the plotted area in the panels (**b**–**d**). Figure generated using QGIS-v3.16.8 (https://www.qgis.org/en/site/).; (**b**) imaging of the cross seismic section; (**c**) seismic horizons identified for the seafloor (dark green) and the BSR (pink); (**d**) calculated heat flow (orange) and temperature at the BSR T_z_ (sky blue) based on assumed lithostatic pressure. Stars indicate the location (**c**) and value (**d**) of the heat flow piston corer measurements along the profile.

**Figure 6:**
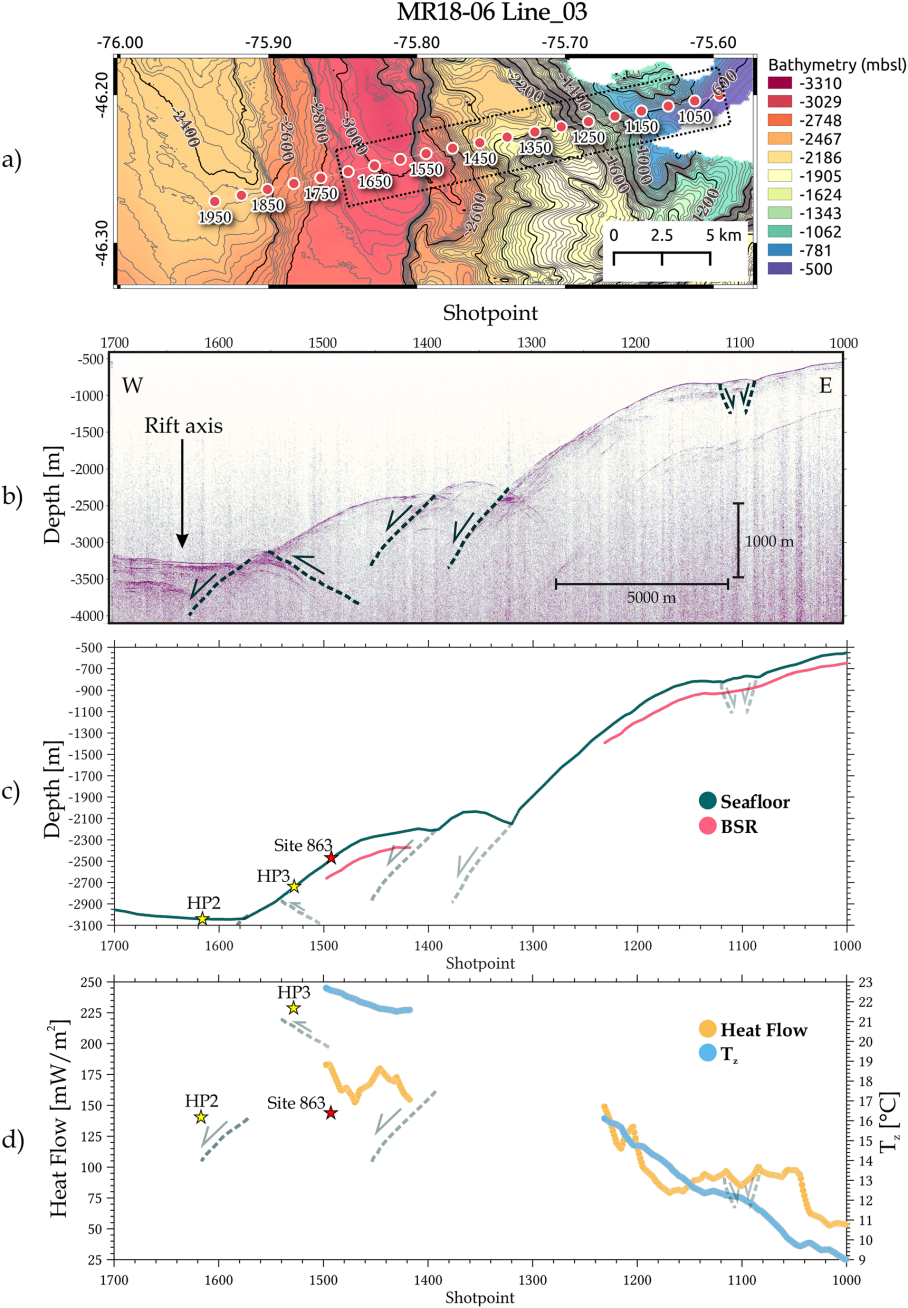
BSR-derived heat flow results from the MR18-06-Line_03 profile: (**a**) location of the seismic profile, red dots indicate the shotpoint values of the Line_03. Dashed rectangle indicates the plotted area in the panels (**b**–**d**). Figure generated using QGIS-v3.16.8 (https://www.qgis.org/en/site/); (**b**) imaging of the cross seismic section; (**c**) seismic horizons identified for the seafloor (dark green) and the BSR (pink); (**d**) calculated heat flow (orange) and temperature at the BSR T_z_ (sky blue) based on assumed lithostatic pressure. Stars indicate the location (**c**) and value (**d**) of the direct heat flow measurements along the profile.

The BSR is continuous, from the lower part of the forearc to up along both seismic sections analysed (Figs. 5 and 6). It only disappears where there is evidence of features of intense deformations, such as faults and fractures that cut sediments reaching the seafloor. Moreover, a very continuous BSR was recognised across the MR18-06 Line_01 profile (Fig. 5b) between 85 and 173 mbsf, whereas a disrupted BSR was recognised across the profile MR18-06 Line_03 (Fig. 6b), with a variable depth of the BSR ranging between 65 and 165 mbsf.

As mentioned in the previous section, the heat flow can be estimated by using the BSR depths. Our results shown in Figs. 5c,d are characterized by higher heat flow values in the northern profile (MR18-06 Line_01), with a maximum value of 285 mW/m^2^ in the lower sector (around shotpoint 1500) that decrease abruptly, around 135 mW/m^2^ to the east, to reach a minimum value of 120 mW/m^2^ in the upper sector.

Similarly, in the southern profile MR18-06 Line_03 (near the trench), a high heat flow value around 183 mW/m^2^ is present, decreasing with a minimum value of 52 mW/m^2^ upwards (Fig. 6c,d). The temperature at the BSR is very high near the trench in both seismic profiles, reaching values around 22 °C that decrease as moving landwards from the trench, with a minimum value around 9 °C at 900 m water depth (Fig. 6d).

### Heat flow piston cores (HP)

Five direct measurement sites were made around the CTJ (see Fig. 2b for an overview), and the heat flow value *q* was calculated as a product of the geothermal gradient G (vertical) and the average shallow (maxima penetration 6 m depth) conductivity *k* as shown in Table 1.

**Table 1:** Heat flow sites in the vicinity of the Chile Triple Junction (MR18-06 cruise report;^75^).

Station	Date	Latitude	Longitude	Water Depth (m)	PEN (m)	G (mK/m)	err (mK/m)	Quality	K (W/m/k)	Q (mW/m^2^)
HP2	Ja n. 17, 2019	46° 14, 726' S	75° 49, 050' W	3040	4	137	0,4	Go o d	1,026	141
HP3	Ja n. 18, 2019	46° 14, 310' S	75° 47, 190' W	2850	2,5	229	0,4	Go o d	1,003	230
HP5	Ja n. 19, 2019	46° 8,995' S	75° 53, 409' W	2809	5	299	0,5	Exc ellent	1,253	375
HP6	Ja n. 19, 2019	46° 8, 799' S	75° 51, 995' W	3054	6	215	0,1	Exc ellent	0,99	213
HP7	Ja n. 19, 2019	46° 7, 799' S	75° 47, 504' W	2278	5	272	0,2	Exc ellent	0,961	261

The highest heat flow value was HP5 = 375 mW/m^2^ in the western flank of the spreading centre on the profile MR18-06 Line_01, whereas the lowest value was HP2 = 141 mW/m^2^ at the trench on the seismic line MR18-06 Line_03.

## Discussion and conclusions

The vicinity of the CTJ is a crucial area for understanding hydrothermal processes and gas hydrate stability in a convergent margin characterized by the subduction of an active spreading centre. It is also a critical window to study, for example, how the continental crust is formed, how seismic coupling is related to geometry, and the nature of the thermal regime of the subducting lithosphere. In the CTJ area, the CR subducts with a low dip beneath a continental margin containing large amounts of methane trapped in hydrate form. In this peculiar subduction zone, a key role in earthquake nucleation and rupture propagation is played by fluids^51^ and they are the main agent of advection heat transfer from depth to the Earth's surface, such as during vigorous fluid advection in the toe of the accretionary prism that can promote hydrate formation at shallow depths on the seabed^6,7,52^.

In this context, a new set of high-resolution bathymetry, five thermal sounding measurements, and two seismic reflection profiles were analysed to study the relationship between the thermal regime, the gas hydrate system, and the geological environment in the vicinity of the CTJ.

### Hydrothermal circulation and features in the western flank of the CR

In relation to direct measurements on the west flank of the ridge, the highest value of heat flow 375 mW/m^2^ (HP5) is observed here, followed by the value of HP6 (213 mW/m^2^, Kinoshita et al. *in prep*). Although HP5 would appear to be “locally high value”, when compared to values at other spreading centres such as the Juan de Fuca ridge with values from 0 to 31,000 mW/m^2^^53^, and Galapagos rift (from 0 to 800 mW/m^2^;^54^), the heat flow value at the CTJ becomes an anomalous “low” value for a spreading centre. This can be explained on one hand by the anomalous large variation in heat flow over a short distance near the spreading centre^53^, and on the other hand the vigorous hydrothermal circulation present can contribute to significant advective heat loss^48^, as was observed in similar studies (e.g.,^55–57^). Note that the HP5 shows higher values of heat flow in accordance with the modelling proposed by^58^, which modelled the hydrothermal flow in correspondence of outcropping fault zones in the mid-Atlantic ridge. This modelling supports the hypothesis that the presence of fault in proximity of HP5 affected the fluid flow increasing the temperature venting compared to surrounding area. Moreover, the low heat flow could be justified by the rapid cooling taking place in this young oceanic lithosphere as already suggested by^59^ on the basis of gravity data acquired in 2009.

**Figure 7:**
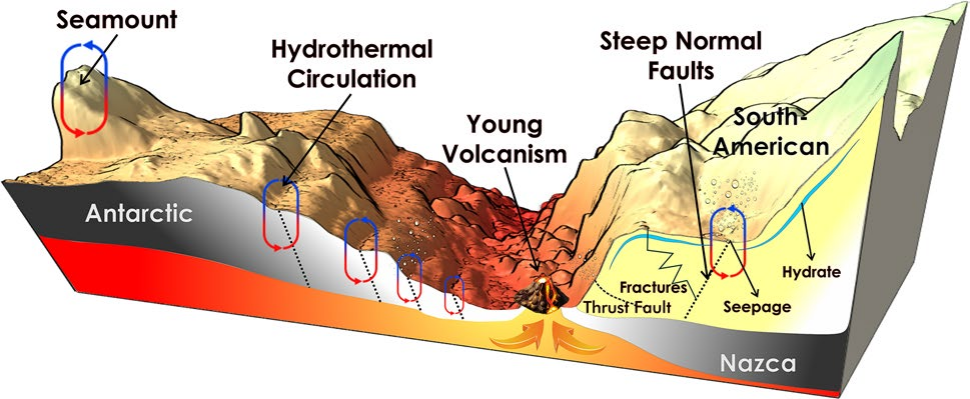
Graphical summary of the study area during the campaign MR18-06 showing the main features that affect the thermal regime at the Chile Triple Junction area. Figure generated using QGIS-v3.16.8 (https://www.qgis.org/en/site/), labels and art were added with the software CorelDRAW Graphics Suite 2021 (https://www.coreldraw.com).

In addition, from the bathymetry, a prominent seamount is observed in the western portion of Fig. 3, which is similar to individual seamounts present near the Iquique Ridge in northern Chile^60^. This pre-subducting seamount may provide high-permeability pathways between the flanks of the basaltic seamount and the overlying ocean, thereby effectively cooling the young oceanic crust (e.g.,^61^). Furthermore, infiltration of large areas of cold seawater and advective extraction of lithospheric heat from the oceanic crust has been observed not only in the vicinity of spreading centres, via flanks or seamount hydrothermal circulation (^62,63^, respectively), but also at the trench-outer rise through extensional bending-related faulting further north of the CTJ (e.g.,^64^). These bending-related faults are associated with the scarp morphology observed on the western flank of the rift axis (Fig. 3b). With all this new information on the western flank of the CR, it is suggested that in this area near the CTJ, where we find these bathymetric features (seamounts and fault-bends, Figs. 2b, 3b, respectively), there is a mixture of factors affecting (cooling) heat flow just prior to subduction as observed in the HP's on the seaward plateau of the axial graben^65^ and summarized in the left part of Fig. 7.

### MR18-06-line_01 profile

From the results of this study, it can be deduced that higher heat flow values (from thermal probes and BSR) are related to the CR's proximity to the spreading centre, in agreement with previous studies in the area^4,6^. The highest heat flow values obtained with both adopted methods are located on the northern seismic profile MR18-06 Line_01 (see Fig. 5). This profile crosses a series of high morphologies (Fig. 2b), described here as young volcanoes because the sample recovered at Site DR19-11 is fresh lava (on top of the “*Kepuche*” volcano; see Supplementary Material I). The fresh glass is likely related to the CR and its associated active volcanism that increases the heat flow in the toe of the continental basement as observed in HP7 (261 mW/m^2^) and BSR-derived heat flow (around shotpoint 1500 in Fig. 5d).

It is important to mention that this chain (from 46°06′ to 46°12′ S) of young volcanoes or axial seamounts with diameter ~ 1 km was described by^50^, and they extend diagonally (NW–SE) from the western flank of the ridge, crossing the trench to the toe of the wedge (Fig. 2b). Two seismic lines, located north and south with respect to the profile MR18-06 Line_01, identified the landward advance of the rift axis, before the collision zone (when it is possible to recognise it on top of the seismic profile) and after the collision zone (when it is already subducted;^23^). For this reason, we deduce that the young volcanoes located in the middle of these two seismic profiles, and located at the toe of the accretionary wedge, correspond to a side of the rift axis. The southernmost volcano is probably very young in age and is likely one of the last remnants of the eastern flank of the rift axis (NP) in an ongoing collision, and because of its uniqueness, we call the volcano "*Kepuche*" ("the last descendant" in the southern Mapuche language).

Iwamori^66^ modelled the distribution of temperature and water during the course of subduction of a young ridge, such as the CR. The results showed that just before the subduction of the ridge, the temperature along the subducting oceanic crust starts to increase and high P/T metamorphism with significant water flux is also reached near the interface when the oceanic crust is younger than 10 Myr. Therefore, we suggest that the local anomalous heat flow at the toe of the wedge is the result of fluid advection encouraged both by the pressure-driven discharge (i.e., dewatering/discharge caused by the horizontal compression of the accreted sediments) and the buoyancy-driven hydrothermal circulation, caused by the subducted active ridge and, probably, by the proximity of the chain of young volcanoes, as illustrated in the right part of Fig. 7. This suggestion makes sense when there is in situ evidence of the presence of two cold seeps in the vicinity of the CTJ, one right at the junction and the other a further 10 km along axis, north of the CTJ, but still within the southernmost segment of the east CR^67^.

Moreover, the BSR-derived heat flow values along the MR18-06 Line_01 seismic profile agree with the values (average of ~ 160 mW/m^2^) of the closest profile RC2901-750 studied by^6^ (see Supplementary Information II), with the exception of the lower part of the wedge (around the HP7 site in Fig. 5d). Here^6^, could not estimate the heat flow in this sector since it was not possible to identify the BSR due to the low resolution of the seismic data. In addition^6^, computed heat flow values near the CTJ, which were much higher than further northwards of the Darwin FZ, with peak values reaching almost 300 mW/m^2^, around the toe of the accretionary wedge. Moreover^6^, estimated high heat flow values (e.g., maximum of 285 mW/m^2^) also in the lower slope of the overriding plate (see Fig. 5e in^6^). These elevated heat flow values suggest that the toe of the wedge is where part of the eastern flank of the CR is currently subducting. In fact, part of the rift axis lies just below the wedge toe^6,23^, where active deformation and vigorous fluid advection are most intense, enhanced by the proximity of the young volcanic chain.

In general, our results of the BSR-derived heat flow values near the toe of the accretionary wedge are much higher than those further up the continental slope (difference of about 100 mW/m^2^) in agreement with previous studies (e.g.,^4,6^). This decrease in heat flow values (see Figs. 5c,d) supports the hypothesis that the Patagonian basement, underlying the upslope portion of the forearc between the Darwin FZ and the CTJ, is not yet being heated by the young subducting oceanic crust^6^.

### Abrupt thermal anomalies

Special attention was devoted to the BSR-derived heat flow value near HP7 (~ 280 mW/m^2^), which drops abruptly by ~ 140 mW/m^2^ over a distance of only 1 km upslope (Fig. 5d). This could be explained by the presence of normal faults in correspondence with the location of HP7 that may serve as a pathway for hot fluids enhanced by the proximity of the young volcanoes at the foot of the wedge (see Fig. 7). Additionally, in the MR18-06 Line_01 profile (Fig. 5d) there are at least four peaks in the heat flow values that are associated with normal faults within the accretionary prism (two peaks near HP7, another at shotpoint ~ 1250 and another at shotpoint ~ 1020 in the shallowest part of the seismic line), agreeing with the assumption that these faults are pathways where hot fluids rise from greater depths and are probably associated with a seepage area on the seafloor.

### Comparison with other studies

**Figure 8:**
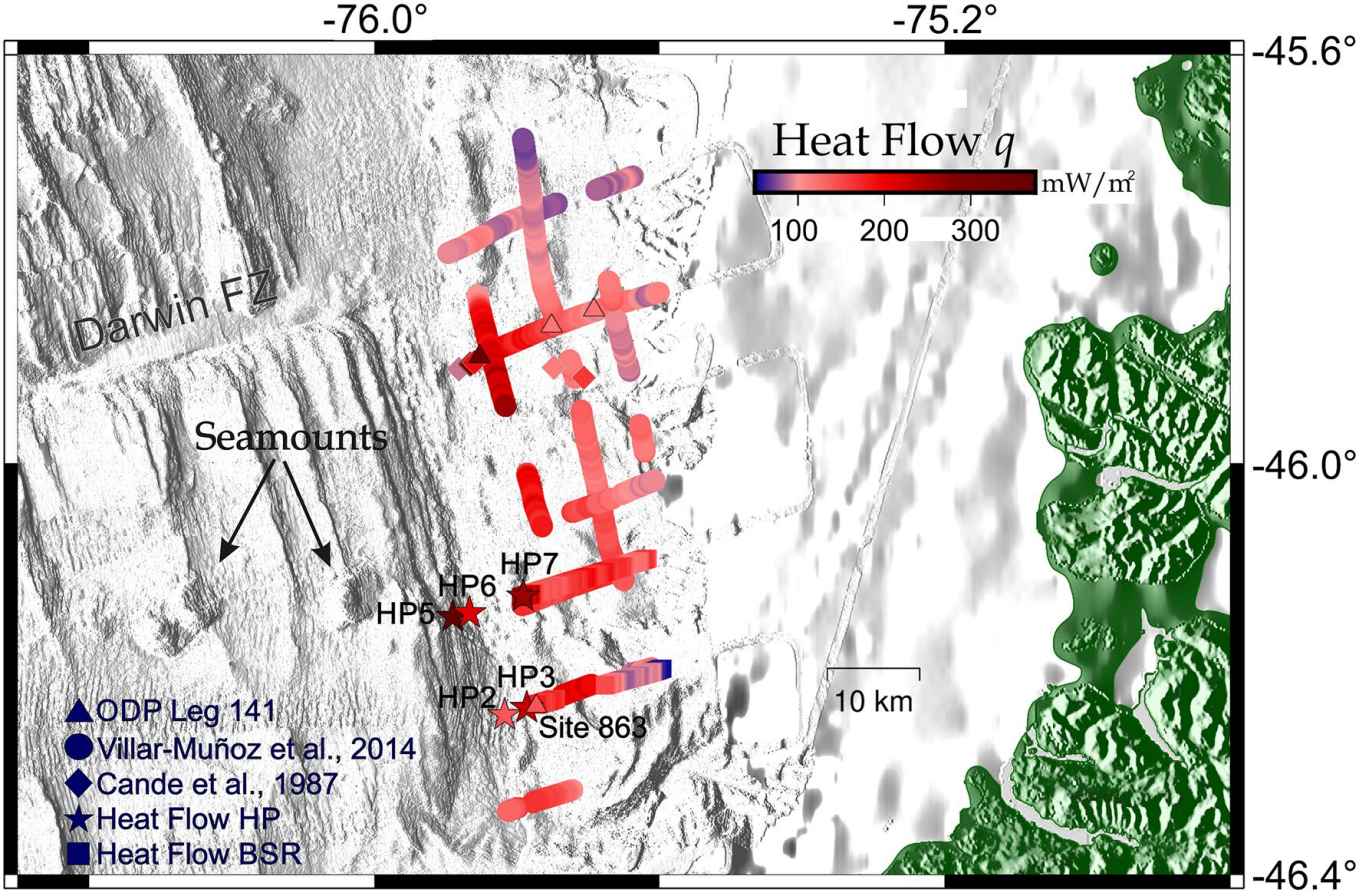
Heat Flow (in mW/m^2^) compilation around the CTJ after^6^: large-scale colour-coded based on BSR-derived heat flow and heat probes available for the area studied. New heat flow Piston core data are displayed as stars, and new BSR-derived heat flow (seismic profiles MR18-06 Line 01 and 03) data are displayed as squares. Figure generated using GMT—v6.2.0 (https://www.generic-mapping-tools.org/).

The agreed value of HP7 (with values estimated from the BSR) is contradicted by some results obtained 30 km north of the CTJ, on the profile RC2901-745, made by^4^ and by^5^, where shallow heat flow values from heat probes were higher than those estimated from the BSR distribution within 5 km of the wedge toe. This difference was attributed to the large hydrogeological activity at the toe (triangles and diamonds close to the trench in Fig. 8). For this reason, we consider that the analysis and comparison of the current low number of seismic profiles and heat probe data are insufficient to obtain a conclusive result, so we strongly recommend further research with greater sampling coverage in the vicinity of the CTJ to understand this system, which has caused major earthquakes and destructive volcanic eruptions in the past.

### MR18-06 line_03 profile

Located further south, which is in correspondence with the RC2901-751 profile described by^6^, a BSR separated by a steep normal fault is identified (see Supplementary Fig. 2b). However, the already subducted rift axis described in other studies is not visualized in our profile due to insufficient energy for shooting and therefore limited seismic resolution. Here, the values of the BSR-derived heat flow decreasing in one half upwards of the fault are similar to those obtained by^6^, providing further evidence that deformation, fracturing, permeability enhancement, and hydrothermal circulation are likely the key agents in facilitating fluid migration from deep strata (e.g., seepage areas) and effectively cooling the wedge (see right part of Fig. 7).

The heat flow value at the toe of the wedge (HP3 = 230 mW/m^2^) in the MR18-06 Line_03 (see Fig. 6d) is higher than the closest estimated values (heat flow BSR-derived = 183 mW/m^2^). The difference between BSR-derived and directly measured heat flows could be related to an over-estimation of the seismic velocity because of the higher amount of fluid along the seismic line RC2901-751^7^, and/or to the HP3 location just above a thrust ridge, an accretionary structure associated with seepage activity (e.g.,^68^) that could increase the heat flow locally. In addition, the HP3 Site is very close to a thrust fault, which has been described in other studies of convergent margins (e.g., Nankai Trough;^69,70^) that may act as permeable pathways for pore fluid expulsion from the wedge and subducted sediments and oceanic crust, causing a scattered heat flow^71^. This suggestion is verified further when considering the warm-fluid supply from deeper sectors, canalised by faults and fractures, reaches the seabed altering the thermal state locally as suggested in previous studies along the Chilean continental margin (e.g.,^6,72^.

In addition, we overlaid in Fig. 6d the value of the Site 863 (ODP 141;^20^), located about 160 m south of Line_03 to see if there is much difference with our BSR-based heat flow results. The value for this site is approximately 140 mW/m^2^, while the corresponding value on Line_03 is close to 180 mW/m^2^. This difference of almost 40 mW/m^2^ can be explained by the distance between the two samples, which may be locally influenced by some kind of hydrothermal circulation that affects the surface heat flow at Site 863, causing the heat flow to vary widely over a short distance near the spreading centers^53^. It may also be that this 20% difference is due to the uncertainty of the method itself (^73,74^; for details see Supplementary Information III).

Finally, it is well known that the Chilean convergent margin has a large deposit of gas hydrates in its margin, and that these are more sensitive to temperature changes which could lead to destabilization. On the other hand, Chile is one of the most active seismic countries in the world and in the CTJ area, several earthquakes from different seismic sources (e.g. intraplate crustal faults, ridge volcanic, interplate) are an additional factor that could affect gas hydrate stability. Therefore, it is essential to implement a database that also includes information on sediment fluids, hydrothermal circulation, seepage areas, etc., to assess the geological risks and environmental hazards associated with a potential massive gas release in this natural laboratory.

## Methods

The data and methods used in this work are based on a multidisciplinary approach to identify and characterize the morphology and estimate the heat flow. In the field and laboratory, theory and modelling were used to reach our objectives. The strategies include bathymetry and seismic reflection processing, heat flow Piston corer in situ measurements and rock sampling.

### Multi-narrow beam bathymetry

The bathymetry used for Figs. 2b, 3, 4, 5, 6 and 7 is a merge between data acquired on board R/V Mirai during the cruise MR08-06 Leg1b in March 2009, and the brand new bathymetry from MR18-06 Leg2 cruise in 2019^75^. The R/V Mirai was equipped with a Multi-narrow Beam Echo Sounding system (MBES), SEABEAM 2112.004 (SeaBeam Instruments Inc.).

### Single-channel seismic reflection profiles

At the CTJ, two new single-channel seismic reflection profiles were acquired during the last geophysical cruise (MR18-06 Leg 2; Fig. 2), and they were used to identify the presence of gas hydrate to estimate the BSR-derived heat flow. The seismic source was composed by a GI gun, with an approximate volume of 150 cubic inches, recorded by a streamer of 24 channels every 3.125 m.

### BSR-derived heat flow

Since the stability of gas hydrates is controlled by pressure P and temperature T conditions^76–78^; therefore, the depth of the base of the gas hydrate layer (or BSR-depth) obtained from the seismic reflection profiles of the expedition MR18-06 were used to calculate the steady-state heat flow q (mW/m^2^) using the formula:

$$ q = k\frac{{T_{z} - T_{0} }}{{\int\limits_{0}^{z} {dz} }} $$

where T_*z*_ and T_*0*_ are the temperatures at the BSR and the seafloor, respectively^79^, *k* is the thermal conductivity, *z* denotes the BSR-depth (in mbsf), and *0* denotes the seafloor (in mbsl). The BSR and seafloor depths were obtained from the seismic profiles MR18-06 Line_01 and Line_03. Seafloor temperatures were taken from CTD measurements (World Ocean Data Base, http://www.nodc.noaa.gov/), and a constant thermal conductivity (*k* = 1.25 W/m K) was used based on the drillcore data ODP Leg 141 Hole 863B^6,20^ to reduce the uncertainty, since the in situ heat flow data are extremely shallow and extrapolating that value to the BSR-depth is not reliable.

The T_*z*_ is calculated by using the gas hydrate dissociation temperature–pressure function^80^:

$$ {1}/{\text{T }} = { 3}.{79 } \times { 1}0 - {3 } - { 2}.{83 } \times { 1}0 - {4 } \times \, \left( {{\text{log}}\,p} \right) $$

where *p* is the pressure (MPa) at the BSR (MPa) and T the temperature (Kelvin). Gas in the system is usually assumed to be pure methane, with a pore water salinity of 35 g/l, supposing a water density equal to 1024 kg/m^3^ and sediment density equal to 2000 kg/m^3^^7,73^.

Hydrostatic pressures and depth can be calculated by converting the measured TWT (two-way travel time) at the BSR using a velocity-depth function derived from the seismic data^81^ or, where available data from ODP, IODP, DSDP drillholes^6,20,52^. To convert water column-TWT into depth, a seawater compressional wave velocity of 1500 m s^−1^ is used; to convert BSR-TWT into depth, it was used a reasonable assumption of the constant velocity value for shallow sediment of 1650 m s^−1^^73^ that fits with the velocity data obtained in the seismic profile RC2901-751 analysed by^7^ close to the CTJ area.

### In situ* heat flow*

One of the objectives of the second leg of MR18-06 was to measure the heat flow at the CTJ^75^. For this purpose, a heat flow Piston corer (HP) was used. It is equipped with 8 temperature loggers for the measurement of the geothermal gradient (G). Sensor holders are made of stainless steel and are designed to protect the temperature logger from the shock at the penetration. The heat flow is obtained as the product of the G and the thermal conductivity (*k*), which was measured on piston core samples using the needle probe method^82^. When the probe penetrates the sediment, it generates frictional heat, increasing the temperature of the temperature loggers. To derive the formation temperature, this frictional heating effect was removed from the data. To obtain a reliable value, it was necessary to measure the sub-bottom temperature for ~ 15 min.

The advantages of this method are that a better (longer) penetration, even into a stiff formation, is reached and that core samples are also collected. On the other hand, the major drawback is that only one penetration per operation is allowed, resulting in high consumption of time and budget.

### Rock sampling

In order to obtain rock and sediment samples from the seafloor, a dredge system (DR) was used during the MR18-06 campaign^75^. Consisting of a chain bag dredge (a box type jaw), handle and steel chain-bag, with box type bucket made from stainless steel (5 mm thick). The DR was settled on and dragged along the ocean bottom, using the A-frame with the lead wire (200 m) and the weight (50 kg × 4–6 blocks).

## Supplementary Information

Supplementary Information 1.

Supplementary Information 2.

Supplementary Information 3.
