# Supplementary material for: A cold seep triggered by a hot ridge subduction
Source: Sci Rep. 2021 Oct 22;11:20923. doi: 10.1038/s41598-021-00414-3 (PMC8536720; doi:10.1038/s41598-021-00414-3)
Supplement: Supplementary file 1 — Supplementary Information 1. [file 41598_2021_414_MOESM1_ESM.pdf]

Supplementary information for "A cold seep triggered by a hot ridge subduction" by Villar-Muñoz et al.

Supplementary Information I

The location of the sample DR19-11 was 46°11.5' S - 75°49.0' W, on top of the southern young volcano "*Kepuche*", with a water depth of 2,903 m (see Fig 2b). The sample shown here was described as a sparsely plagioclase-phyric basalt with a thin quenched glass, presenting a glassy part, pillow rind and slightly vesicular part. None had Mn coating on the surface. More details are described in MR18-06 Cruise Report ([51]; Rock sampling by dredge haul, Orihashi et al.).

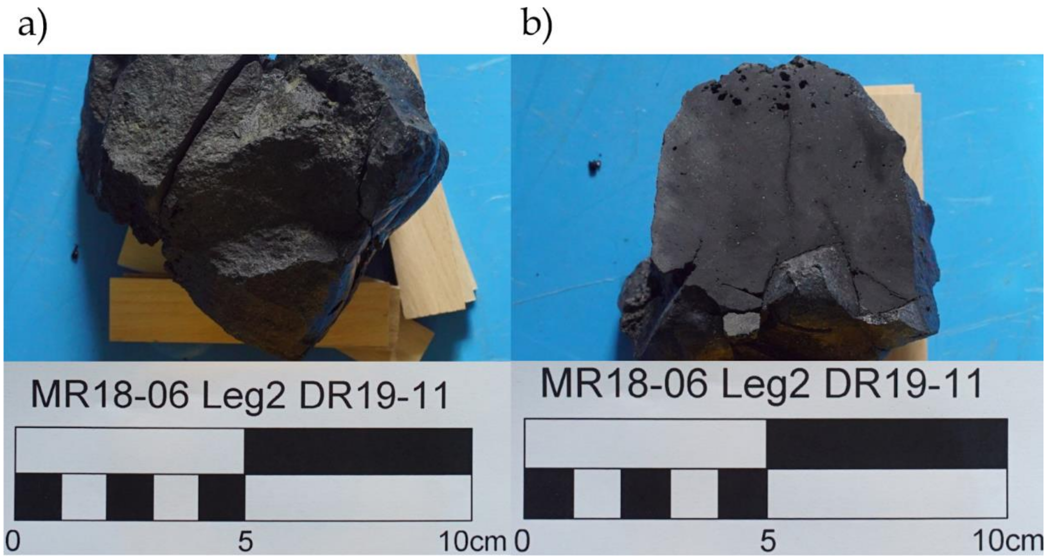

Supplementary Figure 1: Fresh lava sample taken from the southern young volcano at the Chile Triple Junction: a) front section; b) cross-section view.
