# Supplementary material for: A cold seep triggered by a hot ridge subduction
Source: Sci Rep. 2021 Oct 22;11:20923. doi: 10.1038/s41598-021-00414-3 (PMC8536720; doi:10.1038/s41598-021-00414-3)
Supplement: Supplementary file 2 — Supplementary Information 2. [file 41598_2021_414_MOESM2_ESM.pdf]

Supplementary information for "A cold seep triggered by a hot ridge subduction" by Villar-Muñoz et al.

Supplementary Information II

Examples of estimated BSR heat flow after Villar-Muñoz et al., 2014:

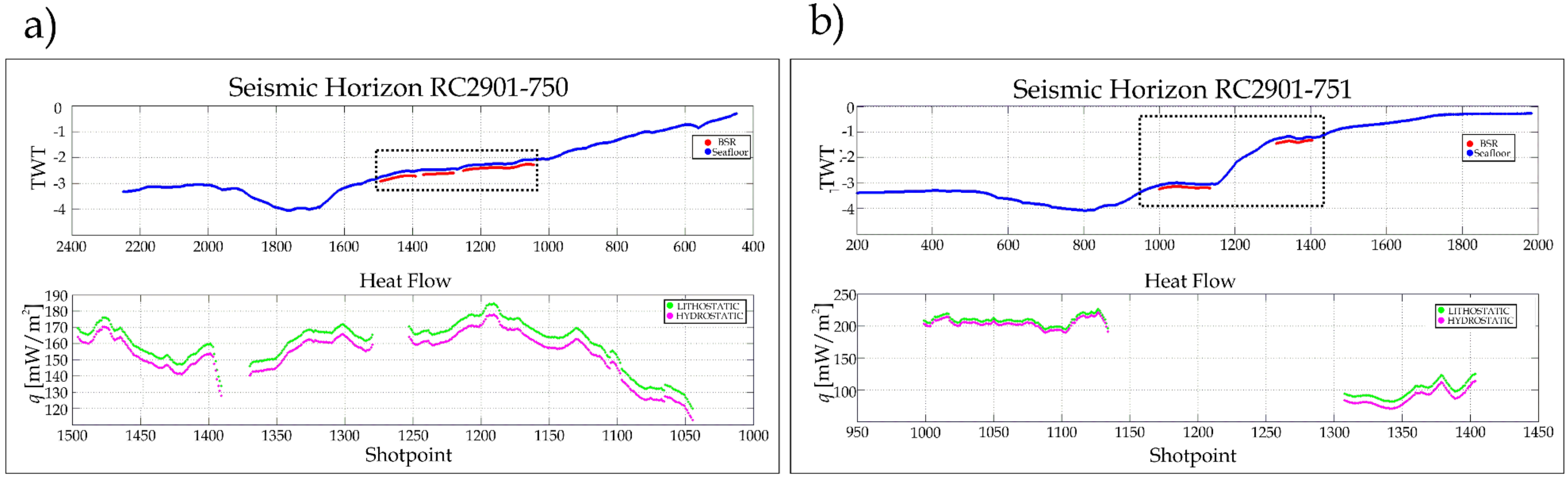

Supplementary Figure 2: a) profile RC2901-750; b) profile RC2901-751. In each diagram, the upper box shows the seismic horizons identified for the seafloor (blue) and the BSR (red), and the lower box the computed heat flow based on assumed lithostatic (green) and hydrostatic pressure (pink).
