# Supplementary material for: A cold seep triggered by a hot ridge subduction
Source: Sci Rep. 2021 Oct 22;11:20923. doi: 10.1038/s41598-021-00414-3 (PMC8536720; doi:10.1038/s41598-021-00414-3)
Supplement: Supplementary file 3 — Supplementary Information 3. [file 41598_2021_414_MOESM3_ESM.pdf]

Supplementary information for "A cold seep triggered by a hot ridge subduction" by Villar-Muñoz et al.

Supplementary Information III

UNCERTAINTIES: The BSR-based estimate in the MR18-06 Line_01 seismic profile agrees well with the value from the heat probe at station HP7 (see Fig. 6d). This indicates that the thermal regime of the zone between the seafloor and the gas hydrate layer (located a few hundred meters below the seafloor) are identical, although both the variables assumed in this study (e.g., conductivity, water/sediment velocities, densities), and the seismic processing and the adopted formulas can have uncertainties. It is important to mention that the heat flow calculations from BSR contain uncertainties on the order of 20% (e.g., [74]) that can be reduced if calibrations by borehole temperature measurements are made. In this study, this uncertainty was reduced by using direct measurements of the thermal conductivity *k* acquired in the ODP141 drillcore, as indicated in the Methods section [20,74].

Due to some uncertain factors, the accuracy of the estimated heat flow values is not very high (uncertainty of ~20% mainly due to ambiguities in the T_z estimation and uncertainties on the thermal conductivity; [75]). Nevertheless, the values are consistent with those measured by conventional methods (e.g., heat probes). Thus, relevant information on the regional distribution of heat flow can be obtained [70].
